# Special Issue: New Advances in Kidney Transplantation

**DOI:** 10.3390/jcm11144190

**Published:** 2022-07-19

**Authors:** Eytan Mor

**Affiliations:** 1 Transplant Center, Department of Surgery B, Sheba Medical Center, Ramat Gan 5266202, Israel; eytan.mor@sheba.health.gov.il; 2 Sackler Medical School, Tel-Aviv University, Tel Aviv 6997801, Israel

This Special Issue in renal transplantation covers a variety of clinical and research areas in kidney transplantation. The recent decade is associated with an ongoing shortage of organs for transplantation with efforts to increase the organ pool with DCDs and extended criteria donors. However, with the increasing success rate of kidney transplants, there is also a growth in the candidate list because of removal of the age barrier and transplantation of high risk patients with other comorbidities. The future seems promising with the development of innovative non-invasive technologies introducing biomarkers for diagnosis of rejection and ischemic reperfusion injury, use of cell therapy for tolerance induction, development of artificial organs, and overcoming immune and non-immune barriers in xenotransplantation. This Special Issue will touch some of these topics that are in the frontiers of the modern era of kidney transplantation.

On the clinical side, there are two papers covering the effect of age and other demographics criteria on long-term outcome after transplant.

The first paper, by Dr Yemini et al., is from my group, and it discusses the “Long-Term Results of Kidney Transplantation in the Elderly: Comparison between Different Donor Settings” [1]. Our paper shows that in a selected population in that age group (>60 y) live-donor transplantation is associated with very good long-term results. As for deceased donor kidney transplantation in the elderly the old-to-old allocation seems to be a rational approach associated with an acceptable outcome. 

In a “Systematic Review and Meta-Analysis on The Impact of Recipient Demographics on Outcomes from Living Donor Kidneys” [2], Dr. Bellini et al. shows that gender mismatch between male recipients and female donors has negative impact on graft survival. African ethnicity and obesity do not influence recipient and graft survival but negatively affect DGF and rejection rates.

As for the effect of immunosuppression on malignancy Dr. Imamura et al. preformed a long term multi-center study showing that “Everolimus Reduces Cancer Incidence and Improves Patient and Graft Survival Rates after Kidney Transplantation” [3]. 

Two other papers focus on pretransplant sensitization. The first paper is by Righini and his colleagues on the “Impact of the Type of Dialysis on Time to Transplantation: Is It Just a Matter of Immunity?” [4]. In that paper, they drew on almost 30 year experience to show that the clinical variables that significantly correlated with longer time to transplantation were the level of presensitization (PRA max and antibodies width) as well as type of dialysis. The lower sensitization rate in the PD population has led to a shorter waiting time until transplant compared to HD group. Another paper, “Apheresis Efficacy and Tolerance in the Setting of HLA-Incompatible Kidney Transplantation” [5] by Dr. Noble and his colleagues, showed that the efficacy of plasmapheresis in lowering preformed anti-HLA antibody levels correlated with the volume of plasma exchanged or filtered and that IA was the most efficient technique for antibody removal compared to plasma exchange (PE) and double filtration plasmapheresis (DFPP). They concluded that apheresis is an effective desensitizing measure that allows kidney transplantation in that high immunological risk group of patients.

Use of biomarkers in transplantation is another innovative area of interest in recent years. In this Special Issue there are two papers looking at correlation of biomarker and outcome after transplant. The first one is “Pretransplant Serum Uromodulin and Its Association with Delayed Graft Function Following Kidney Transplantation” [6]. In that paper, Dr. Kemmner et al. evaluated the association between serum uromodulin (sUMOD), a potential marker for tubular integrity, with DGF in the clinical setting. They report that higher pretransplant sUMOD was independently associated with lower odds for DGF, potentially serving as a non-invasive marker to stratify patients according to their risk for developing DGF early in the setting of kidney transplantation. The second paper is on the use of “Urinary NGAL Measured after the First Year Post Kidney Transplantation to Predict Changes in Glomerular Filtration over One-Year Follow-Up” [7] by Dr. Keilar and her colleagues. In the clinical setting, we are using biochemical markers in the blood (creatinine levels) and urine (albumin and protein levels) to assess graft function late after transplant. Introduction of new and more sensitive markers are needed in stable patient who may develop quiescent graft injury. In their study, Dr. Keilar and her colleagues assessed the urinary concentrations of neutrophil gelatinase-associated lipocalin (NGAL) as a predictor of changes in kidney transplant function after the first year after transplantation among 109 patients with stable functioning graft. They found that Urinary NGAL measured at baseline was twice higher in patients with at least 10% decrease in eGFR over 1-year follow-up compared to those with stable or improving transplant function. Baseline NGAL significantly predicted the relative and absolute changes in eGFR.

The last few years have seen the emergence of many new technologies designed to examine organ function, including new imaging techniques, transcriptomics, genomics, proteomics, metabolomics, lipidomics, and new solutions in organ perfusion, which has enabled a deeper understanding of the complex mechanisms associated with ischemia-reperfusion injury (IRI), inflammatory process, and graft rejection. This issue includes “A Review of Current and Emerging Trends in Donor Graft-Quality Assessment Technique” [8] written by Ms. Natalia Warmuzińska and her colleagues that summarizes and assesses the strengths and weaknesses of current conventional diagnostic methods and a wide range of new potential strategies with respect to donor graft-quality assessment, the identification of IRI, perfusion control, and the prediction of DGF. One of the new methods to assess graft quality is described in another paper by Dr. lau et al. who used “Intraoperative Near-Infrared Spectroscopy Monitoring of Renal Allograft Reperfusion in Kidney Transplant Recipients” [9]. In their study they used a handheld near-infrared spectroscopy (NIRS) device to quantify regional tissue oxygen saturation levels (rSO_2_) in the renal allograft after reperfusion and compared the rSO_2_ between recipients of a deceased donor and a living donor. They showed that rSO_2_ remained significantly lower in the DDRT group compared to the LDRT group throughout the 50 min after reperfusion and that reperfusion rates were significantly faster in the LDRT group during the first 5 min post-reperfusion. Interestingly, intraoperative rSO_2_ strongly correlated with allograft function up to 14 days post-transplantation. They concluded that NIRS may be a useful intra-operative tool to assess the degree of preservation/reperfusion injury and predict early allograft function.

Lastly, future technologies to develop organs to replace the current source of human organs for transplant are in the focus of many research groups around the world. Aiming to achieve future generation of a new kidney Dr. Garcia-Dominguez and his colleagues studied “The effect of Sildenafil Citrate in enhancing renal organogenesis following metanephroi allotransplantation” [10]. Sildenafil citrate (SC) is known as a useful inductor of angiogenesis, offering renoprotective properties due to its anti-inflammatory, antifibrotic, and antiapoptotic effects. In their animal model Dr. Garcia-Dominguez and his colleagues using an animal model performed metanephroi allotransplantation after embedding sildenafil citrate into the retroperitoneal fat. After 21 days the new kidneys’ weights become increased significantly. Functionality was proven by renin and erythropoietin gene expression and tubular integrity was evident by highly expressed E-cadherin on Immunofluorescence assay. Histological studies showed mature glomeruli and hydronephrosis showing the new kidney’s excretory function.
